# *Drosophila cyfip* Regulates Synaptic Development and Endocytosis by Suppressing Filamentous Actin Assembly

**DOI:** 10.1371/journal.pgen.1003450

**Published:** 2013-04-04

**Authors:** Lu Zhao, Dan Wang, Qifu Wang, Avital A. Rodal, Yong Q. Zhang

**Affiliations:** 1 Key Laboratory of Molecular and Developmental Biology, Institute of Genetics and Developmental Biology, Chinese Academy of Sciences, Beijing, China; 2 Department of Entomology, China Agricultural University, Beijing, China; 3 Department of Biology, Brandeis University, Waltham, Massachusetts, United States of America; Stanford University School of Medicine, United States of America

## Abstract

The formation of synapses and the proper construction of neural circuits depend on signaling pathways that regulate cytoskeletal structure and dynamics. After the mutual recognition of a growing axon and its target, multiple signaling pathways are activated that regulate cytoskeletal dynamics to determine the morphology and strength of the connection. By analyzing *Drosophila* mutations in the cytoplasmic FMRP interacting protein Cyfip, we demonstrate that this component of the WAVE complex inhibits the assembly of filamentous actin (F-actin) and thereby regulates key aspects of synaptogenesis. Cyfip regulates the distribution of F-actin filaments in presynaptic neuromuscular junction (NMJ) terminals. At *cyfip* mutant NMJs, F-actin assembly was accelerated, resulting in shorter NMJs, more numerous satellite boutons, and reduced quantal content. Increased synaptic vesicle size and failure to maintain excitatory junctional potential amplitudes under high-frequency stimulation in *cyfip* mutants indicated an endocytic defect. *cyfip* mutants exhibited upregulated bone morphogenetic protein (BMP) signaling, a major growth-promoting pathway known to be attenuated by endocytosis at the *Drosophila* NMJ. We propose that Cyfip regulates synapse development and endocytosis by inhibiting actin assembly.

## Author Summary

Synapses are specialized junctions at which neurons communicate with target cells. To establish properly wired neuronal circuits, synapses must grow in size and strength with a high degree of accuracy. The actin cytoskeleton plays a crucial role in the formation and function of synapses, but the underlying mechanisms remain poorly understood. The *Drosophila* neuromuscular junction (NMJ) is an excellent model for studying synaptic development and function. By analyzing *Drosophila* mutants of the cytoplasmic FMRP interacting protein Cyfip, we establish that this protein inhibits the assembly of filamentous actin (F-actin). At *cyfip* mutant NMJ synapses, F-actin assembly was accelerated and NMJ terminals were shorter and grew supernumerary buds. Furthermore, neurotransmission was not sustained under high-frequency stimulation. These changes could be caused by defects in synaptic endocytosis, which would compromise the endocytic attenuation of signaling pathways such as the NMJ growth-promoting bone morphogenetic protein (BMP) pathway. Indeed, BMP signaling was upregulated in *cyfip* mutants. We propose that Cyfip regulates synaptic development and function by inhibiting F-actin assembly, which in turn downregulates BMP signaling via endocytosis. This study establishes a novel role for Cyfip-mediated regulation of the actin cytoskeleton at the *Drosophila* NMJ.

## Introduction

To establish functional neural circuits, synapses must form at specific locations and grow to an appropriate size and strength. A multitude of signaling pathways are required to achieve and maintain these precise patterns of synaptic connectivity [1]–[3]. Many of these signals regulate local actin cytoskeletal networks, which are crucial for both synapse formation and plasticity [4]–[6]. Precisely how the actin cytoskeleton integrates various signaling pathways to regulate synaptic formation and function remains to be elucidated.

At *Drosophila* neuromuscular junctions (NMJs), dysregulation of actin dynamics results in morphological defects, including the formation of excess satellite boutons. For example, mutants of the actin regulator nervous wreck (Nwk), an N-WASP (neuronal Wiskott–Aldrich syndrome protein) interacting protein, show excess satellite boutons at NMJs [7]. Nervous wreck activates WASP-Arp2/3-mediated actin polymerization and coordinates with Cdc42 to regulate actin assembly [8]. Additional actin regulatory proteins implicated in synapse formation include WASP, spectrin, and adducin [6], [9], [10]. Moreover, these proteins and their interactors are conserved across species, indicating a seminal role for the actin cytoskeleton in synaptic development.

In addition to regulating synaptic development, multiple lines of evidence show that actin and its regulators function in synaptic endocytosis. First, filamentous actin (F-actin) is observed around synaptic vesicle clusters where it facilitates vesicle endocytosis or mobility [11], [12]. Second, many actin regulators bind endocytic proteins directly or indirectly. For example, Cdc42, WASP, and Nwk all interact directly with the endocytic protein intersectin-1/Dap160, an important binding partner of dynamin [7], [8], [13]. Third, disruption of the actin cytoskeleton impairs vesicle recycling at both vertebrate and invertebrate synapses [12], [14]. Fourth, actin regulator mutants such as *twinfilin*, *dap160/intersectin*, and *nwk* show defects in synaptic endocytosis [7], [15]–[17]. In addition to endocytosis of synaptic vesicle membrane, receptors must be retrieved from the presynaptic membrane to downregulate specific signaling pathways. At the *Drosophila* NMJ for example, actin-mediated endocytosis downregulates the bone morphogenetic protein (BMP) signaling pathway that normally promotes synaptic growth [1], [7], [8], suggesting that actin cytoskeleton may contribute to synaptic development by regulating endocytosis.

The heteropentameric WAVE complex, composed of WAVE (WASP/verprolin homologous protein), Cyfip/Sra-1/Pir121, Kette/Nap1, Abi, and HSPC300 [18]–[21], relays signals from the Rac GTPase to the actin nucleator Arp2/3 complex to control de novo F-actin assembly. The organization of the WAVE complex is well established in vitro. Specifically, Abi and Nap1 form the core sub-complex and Cyfip binds Nap1, while both WAVE and HSPC300 bind the N-terminus of Abi [20], [21]. In the resting state, the verprolin-homology, central, and acidic (VCA) domain of the WAVE protein is sequestered by binding to Cyfip and/or Nap1 [18], [21]. Upon Rac1 binding to the N-terminus of Cyfip, together with other coincident signals, the VCA domain is released from the WAVE complex to activate the actin nucleator Arp2/3 [18], [21]–[23]. However, this transduction mechanism has only been demonstrated in vitro, while the exact role of each component in regulating the activity of the WAVE complex in vivo is poorly understood.

We provide evidence that loss of Cyfip leads to enhanced F-actin assembly in *Drosophila*, resulting in altered NMJ morphology. We also report that Cyfip loss disrupts synaptic endocytosis, likely by regulating presynaptic F-actin networks. The bone morphogenetic protein (BMP) signaling attenuated by endocytosis is upregulated in *cyfip* mutants, consistent with an endocytic defect. Reducing the level of SCAR, the *Drosophila* homolog of WAVE, partially rescues the morphological anomalies, endocytic defects, and enhanced actin dynamics at *cyfip* mutant NMJs. Thus, our findings demonstrate that Cyfip regulates synapse formation and endocytosis by inhibiting actin dynamics.

## Results

### Cyfip regulates NMJ development

*Drosophila cyfip^85.1^* null mutants were early pupal lethal, consistent with a previous report [24]. Tissue-specific expression of Cyfip in muscles driven by *C57-Gal4* or in neurons driven by *elav-Gal4* allowed survival of *cyfip^85.1^* nulls to late pupal stages, but no adults emerged (Table 1). In contrast, 85.63% of homozygous *cyfip^85.1^* nulls ubiquitously expressing Cyfip under the control of *act-Gal4* survived to adulthood. Thus, the lethality of *cyfip* nulls was specifically caused by loss of Cyfip.

**Table 1:** Rescue of *cyfip* mutant lethality by tissue-specific expression of Cyfip.

	Late pupal viability (%)	Adult viability (%)	Numbers scored
*Cyfip^85.1^/TM6B*	4	0.0	928
*C57-Gal4 cyfip^85.1^/TM6B* X *UAS-cyfip; cyfip^85.1^/TM6B*	100	0.0	818
*elav-Gal4*; *cyfip^85.1^/TM6B* X *UAS-cyfip*; *cyfip^85.1^/TM6B*	100	0.0	938
*act-Gal4 cyfip^85.1^*/*TM6B* X *UAS-cyfip*; *cyfip^85.1^*/*TM6B*	100	85.63	497

The percentage viability is shown as the number of progeny survived to late pupal stages or adults compared to the expected viabilities that should be one half of the balancer siblings. Expression of Cyfip driven by different Gal4 drivers muscular *C57-Gal4*, pan-neuronal *elav-Gal4*, and ubiquitous *act-Gal4* rescued the lethality to different stages, depending on the tissue-specific Gal4 used.

**Figure 1:**
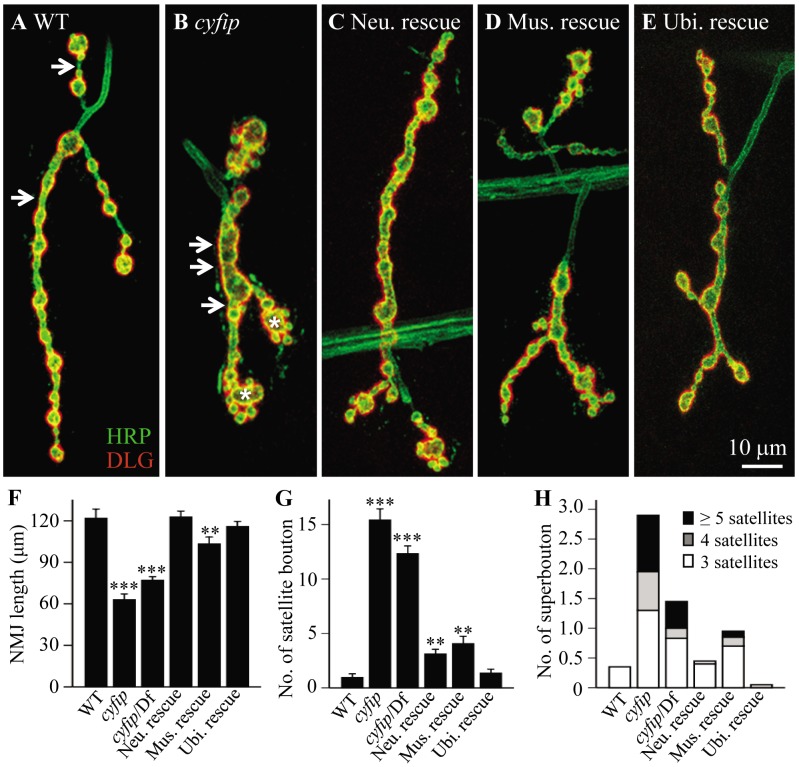
*cyfip* regulates synapse development. (A–E) Representative NMJ4 synapses from different genotypes co-stained with anti-HRP recognizing the neuronal plasma membrane (green) and an antibody against DLG (red), a postsynaptic scaffold protein. Arrows indicate interbouton spacing; asterisks denote superboutons with multiple satellite boutons attached. Scale bar, 10 µm. (F–H) Statistical results of NMJ length (F), the number of satellite boutons (G), and the number of superboutons (H) in different genotypes. Ubiquitous expression of *cyfip* controlled by *act-Gal4* fully rescued the NMJ phenotypes, whereas pre- and postsynaptic expression of *cyfip* by *elav-Gal4* and *C57-Gal4*, respectively, partially rescued the satellite bouton phenotype of *cyfip^85.1^* null mutants. *n* = 20 for each genotype; * *p*<0.05, *** *p*<0.01, *** *p*<0.001; error bars indicate SEM.

The muscle 4 NMJ of wild-type (*w^1118^*) wandering third instar larvae shows a stereotyped “beads-on-a-chain” pattern of boutons (Figure 1A). In contrast to the wild-type controls, NMJ boutons in *cyfip^85.1^* null homozygotes appeared clustered together with little or no spacing between them (Figure 1B). The total NMJ length was reduced by 50% in *cyfip^85.1^* mutants relative to wild type (*p*<0.001; Figure 1A, 1B, and 1F), in agreement with a previous report [24]. *cyfip* hemizygous mutants in which *cyfip^85.1^* was on one chromosome, while *Df(3R)Exel6174*, which uncovers *cyfip*, was on the other also showed reduced NMJ length (*p*<0.001; Figure 1F). Muscle expression of Cyfip partially rescued, while neuronal or ubiquitous expression of Cyfip fully rescued the short NMJ phenotype of *cyfip^85.1^* mutants (Figure 1F).

Small ectopic boutons that bud from synaptic branches or primary boutons are normally rare and referred to as satellite boutons [7], [25], [26]. At *cyfip^85.1^* homozygous mutant NMJ4, there was a 15-fold increase in the number of these satellite boutons (*p*<0.001; Figure 1A, 1B, and 1G), described previously as “supernumerary buds” [24]. *cyfip* hemizygotes (*cyfip^85.1^*/*Df(3R)Exel6174*) showed a similar increase in the number of satellite boutons (*p*<0.001; Figure 1G). Neuronal or muscular expression of Cyfip partially rescued the phenotype, as there was still a 3- or 4-fold increase in the number of satellite boutons compared to wild-type controls (Figure 1C, 1D, and 1G), whereas ubiquitous expression of *cyfip* fully rescued the excess satellite bouton of *cyfip^85.1^* nulls (Figure 1E and 1G). The satellite boutons form primarily in the late larval stages of *cyfip* mutants (Figure S1). These phenotypes were highly penetrant, as other NMJ terminals such as NMJ6/7 were also shorter and exhibited excess satellite boutons.

We then quantified the number of superboutons, defined as parental primary boutons from which at least three satellite boutons formed. The average number of superboutons was significantly higher in *cyfip^85.1^* homozygous mutants (2.9±0.27 per NMJ4) compared to wild type (0.35±0.13) (Figure 1H; *p*<0.001). Additionally, nearly 30% of superboutons in mutants possessed five or more satellite boutons (Figure 1H). Again, ubiquitous expression of Cyfip fully rescued, while neuronal or muscular expression partially rescued the superbouton phenotype of *cyfip^85.1^* mutants (Figure 1C–1E and 1H). These results demonstrate that *cyfip* regulates synapse formation from both pre- and postsynaptic sides.

### *cyfip* mutants display enlarged synaptic vesicles at active zones and more presynaptic cisternae

**Figure 2:**
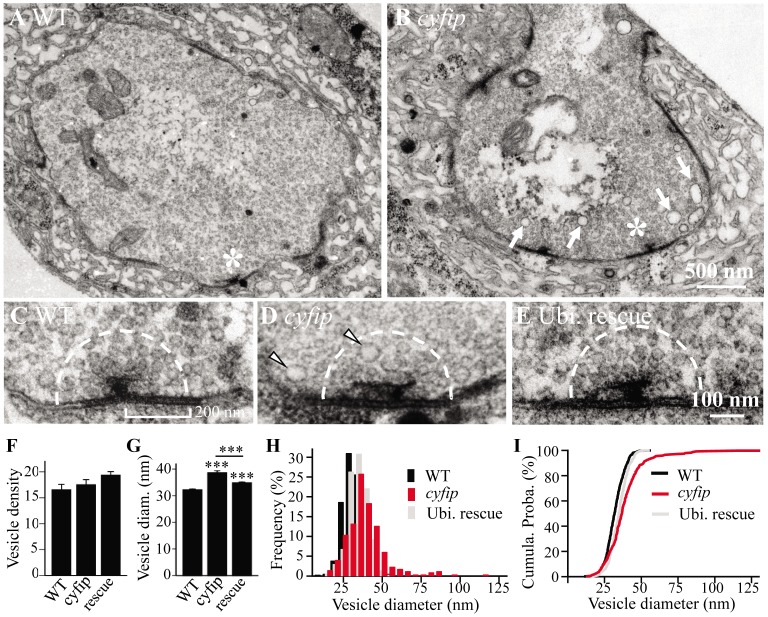
Enlarged synaptic vesicles at active zones and more cisternae in the synaptic boutons of *cyfip* mutants. (A, B) Electron micrographs of synaptic boutons from wild type (A) and *cyfip^85.1^* mutants (B). Asterisks indicate active zones with T bars. Compared to wild type, *cyfip^85.1^* mutants exhibited significantly more cisternae (arrows in B). Scale bar, 500 nm. (C–E) High magnification view of representative active zones in wild type (C), *cyfip^85.1^* mutants (D), and ubiquitous expression of Cyfip driven by *act-Gal4* in *cyfip^85.1^* background (E). Arrowheads in (D) indicate enlarged vesicles near the T-bar. Dashed line defines a 200 nm radius around the active zone for quantitative analysis of SVs. (F, G) Quantification of the number (F) and diameter (G) of SVs within a 200 nm radius of the active zone. *n* = 446 SVs for wild type, *n* = 350 SVs for mutants, and *n* = 445 SVs for the rescue. (H, I) Frequency distributions (H) and cumulative probabilities (I) of SV diameters in the defined area around the active zone.

To understand the synaptic defects in *cyfip* mutants at the ultrastructural level, we examined NMJ synapses in wandering third instar larvae by electron microscopy. Many features, such as the number and structure of mitochondria, the number and morphology of active zones, and vesicle density in the whole bouton appeared largely normal in *cyfip^85.1^* mutants compared to wild-type controls (Figure 2A and 2B). The mean number of synaptic vesicles (SVs) within a 200 nm radius of the active zone was also normal (*n*≥20; *p*>0.05), but the mean SV diameter was 19.8% larger in *cyfip^85.1^* mutants (*p*<0.001; Figure 2C, 2D, and 2G). Ubiquitous expression of Cyfip by *act-Gal4* partially but significantly rescued the increased SV diameter in *cyfip^85.1^* mutants (Figure 2E and 2G). Histograms and cumulative probability plots of the SV size distribution showed that 89% of wild-type SVs and 80% of SVs in rescued animals were <40 nm, whereas only 65% of *cyfip* mutant SVs were <40 nm in diameter (Figure 2H and 2I). In addition, the number of cisternae (presumably endosomes) >60 nm in diameter per bouton cross-section was dramatically higher in mutants (arrow in Figure 2B). Enlarged SVs and more cisternae have been reported in mutants with deficient endocytosis, including *AP180*/*lap*, *dap160*, *eps15*, *tweek*, and *flower* mutants [15], [27]–[30], suggesting that *cyfip* may regulate endocytosis and/or vesicle recycling.

### Increased quantal size and decreased quantal content at *cyfip* mutant NMJs

**Figure 3:**
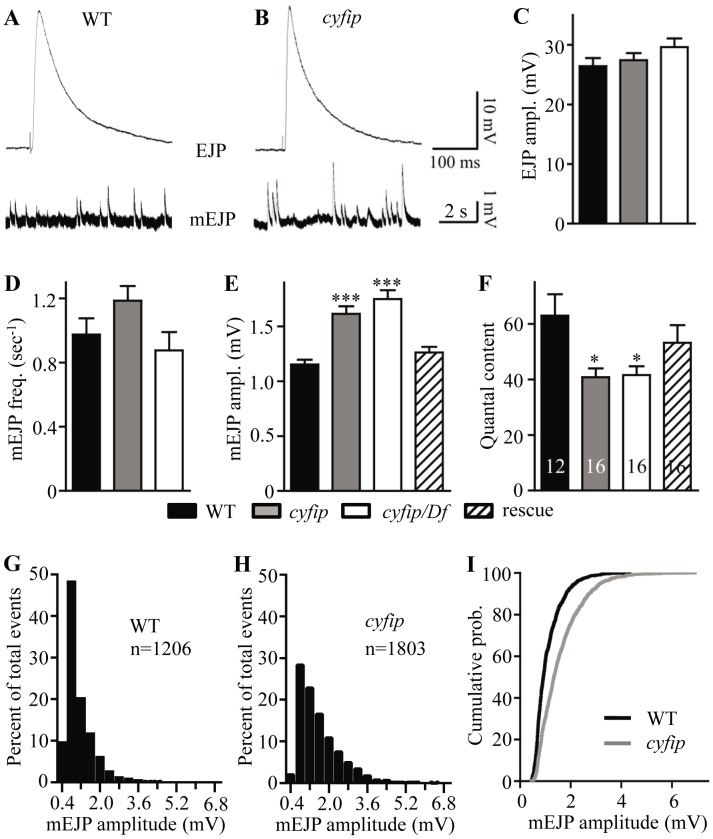
*cyfip* mutants show normal evoked junctional potential (EJP) amplitudes but larger spontaneous mEJP amplitudes. (A, B) Representative excitatory junctional potentials (EJPs) and spontaneous miniature EJPs (mEJPs) recorded from wild type and *cyfip^85.1^* mutant NMJs. (C–F) Statistical analysis of mean EJP amplitude (B), mEJP frequency (C), mEJP amplitude (D), and quantal content (E) in different genotypes. The mEJP amplitude is significantly higher whereas the quantal content is lower in *cyfip^85.1^* mutants compared to wild type. The number of animals analyzed is indicated in (F); * *p*<0.05, *** *p*<0.001; error bars indicate SEM. (G–I) Frequency distributions (G, H) and cumulative probabilities (I) of mEJP amplitudes recorded from wild type and *cyfip^85.1^* mutants.

To determine if synaptic transmission was impaired in *cyfip* mutants, we stimulated the motor neurons and recorded excitatory junctional potentials (EJPs) from NMJ6/7 in abdominal segments A2 or A3 of wandering third instar larvae. We first recorded evoked EJPs at 0.3 Hz in a modified HL3 saline containing 0.23 mM Ca^2+^. At this Ca^2+^ concentration, the EJP amplitudes of wild-type controls and *cyfip^85.1^* mutants were similar (Figure 3A–3C).

We then analyzed quantal size by recording the amplitudes of miniature EJPs (mEJPs). Mean mEJP amplitudes were 40% larger in both homozygous and hemizygous *cyfip^85.1^* mutants compared to wild type (*p*<0.001; Figure 3A, 3B, and 3E), whereas the mEJP frequency in *cyfip^85.1^* mutants was not altered (Figure 3A, 3B, and 3D). The increased quantal size in *cyfip^85.1^* mutants was fully rescued by presynaptic expression of *cyfip* driven by *elav-Gal4* (Figure 3E). Histogram and cumulative probability plot of mEJP amplitudes revealed that large-amplitude mEJPs occurred more often in *cyfip^85.1^* mutants than in wild type. Specifically, mEJP amplitudes larger than 4 mV occurred in *cyfip^85.1^* mutants but were never seen in wild type (Figure 3G–3I). Quantal size is closely correlated with vesicle size, a likely determinant of transmitter content per vesicle [15], [30]. As there were no changes in the expression of postsynaptic glutamate receptors detected by immunostaining (data not shown), the increased quantal size suggests that the enlarged SVs in *cyfip* mutants release more neurotransmitter.

The normal EJP amplitude and increased quantal size in *cyfip* mutants indicate that the number of vesicles released per stimulus (quantal content) was reduced by 35% compared to wild type (*p*<0.05; Figure 3F). The reduced quantal content in *cyfip^85.1^* mutants was rescued to the wild-type level by presynaptic expression of Cyfip controlled by *elav-Gal4* (Figure 3F). The reduced quantal content may arise from a homeostatic control mechanism that compensates for the abnormally enlarged vesicles to maintain a constant synaptic output.

### Cyfip is required for SV recycling

**Figure 4:**
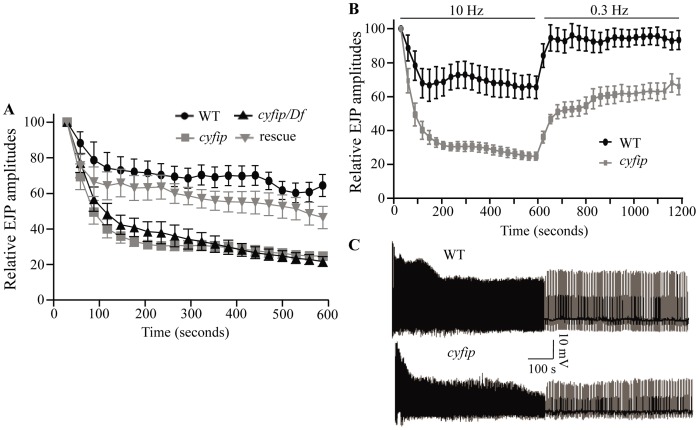
*cyfip* mutants fail to sustain normal neurotransmitter release during high-frequency stimulation. (A) Statistical analysis of EJP amplitudes under 10 Hz stimulation for 10 min. Comparison of EJPs from wild type (WT), *cyfip^85.1^* (*cyfip*), *cyfip^85.1^/Df(3R)Exel16174* (*cyfip/Df*), and *cyfip^85.1^* mutants expressing Cyfip presynaptically driven by *elav-Gal4* (rescue) reveals faster rundown of EJP amplitudes in *cyfip^85.1^* and *cyfip^85.1^/Df* mutants compared to wild type. (B) Amplitudes of EJPs during tetanic stimulation at 10 Hz for 10 min, and then during low-frequency stimulation at 0.3 Hz for 10 min. *cyfip^85.1^* mutants displayed a significantly slower recovery of EJP amplitudes. *n* = 10 for each genotype. Error bars indicate SEM. (C) Representative EJP traces recorded from wild type and *cyfip^85.1^* mutants.

The increased number of satellite boutons, the enlarged SVs, and the increased mEJP amplitudes (Figure 1, Figure 2, Figure 3) in *cyfip^85.1^* mutants all indicate a possible role for Cyfip in endocytosis. If so, then *cyfip^85.1^* mutant synapses may be more prone to EJP rundown during high-frequency stimulation. During a 10 min train of 10 Hz stimulation in modified HL3 saline containing high Ca^2+^ (10 mM), wild-type synapses exhibited a slow EJP decline to 65.4±6.6% of the initial amplitude (Figure 4A). In homozygous *cyfip^85.1^* nulls, however, EJPs evoked by the same 10 Hz stimulus train exhibited a much faster decline within the first 120 s and decreased to 24.8±2.4% of the initial amplitude by the end of stimulation (Figure 4A). Hemizygous *cyfip^85.^* ^1^/*Df(3R)Exel6174* mutants also displayed an enhanced rundown in EJP amplitude similar to that of homozygous mutants. Furthermore, neuronal expression of *cyfip* driven by *elav-Gal4* largely reversed the faster decline in EJP amplitude observed in homozygous *cyfip^85.1^* null mutants, indicating that this phenotype is specifically caused by *cyfip* loss-of-function. The enhanced rundown of EJPs shows that *cyfip* participates in the replenishment of SVs.

If endocytosis and concomitant membrane recycling were compromised, then the NMJ vesicle pool would not recover as quickly as wild type to the prestimulus level after depletion [31]. We stimulated the nerve at 0.3 Hz for 10 min following the 10 min tetanic stimulation to measure recovery from synaptic depletion. Indeed, EJP amplitudes in wild-type larvae recovered within 60 s to reach 94.2±7.7% of the prestimulus EJP amplitude during the low-frequency (0.3 Hz) stimulus train (Figure 4B and 4C), while EJP amplitude at *cyfip^85.1^* NMJs reached only 65.9±4.8% of the prestimulus level after 10 min of 0.3 Hz stimulation (Figure 4B and 4C). The enhanced rundown and slower recovery of EJP amplitudes in *cyfip^85.1^* mutant NMJs strongly suggest that Cyfip is required for normal vesicle recycling.

### Excess satellite boutons in *cyfip* mutants result from upregulated BMP signaling

The excess satellite boutons in *cyfip* mutants likely result from dysregulation of signaling pathways that modulate NMJ growth. Accumulating evidence demonstrates that retrograde BMP signaling is the primary growth-promoting pathway at developing *Drosophila* NMJs [1], [7]. Given that the supernumerary satellite bouton phenotype of *cyfip^85.1^* mutants resembled that observed in animals with upregulated BMP signaling [7], we examined if BMP signaling was over-activated in *cyfip* mutants.

**Figure 5:**
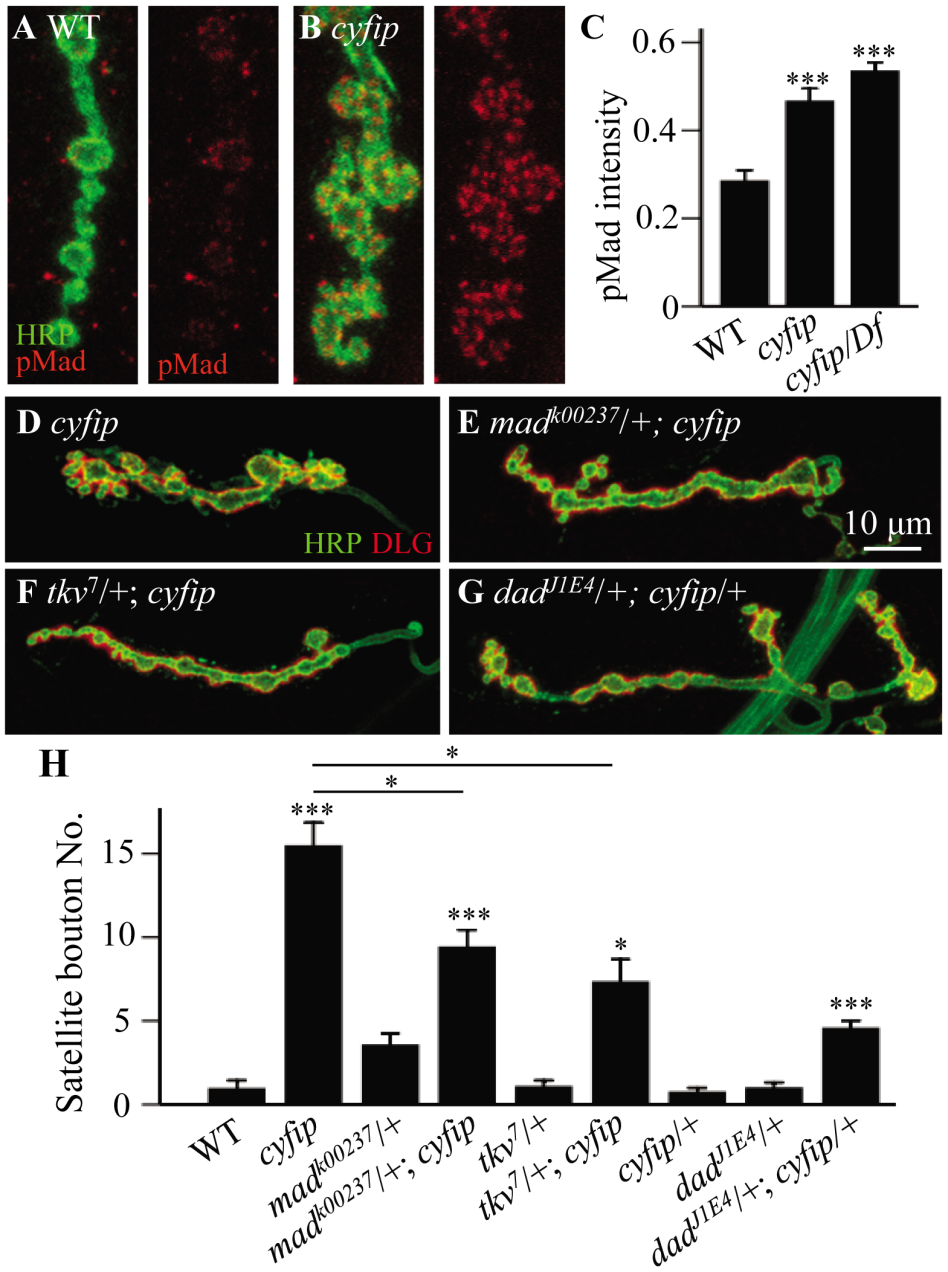
The excess satellite bouton formation in *cyfip* mutants depends on elevated BMP signaling. (A, B) Representative synaptic boutons labeled with anti-HRP (red) and anti-pMad (red) in wild type (A) and *cyfip^85.1^* mutants (B). pMad level was increased in *cyfip^85.1^* mutant synapses. Scale bar, 5 µm. (C) Statistical analysis of pMad staining intensity. *n*≥10 for each genotype. (D–G) Representative NMJ4 synapses double-labeled with anti-HRP (red) and anti-DLG (red) from *cyfip^85.1^* (D), *mad^k00237^/+*; *cyfip^85.1^* (E), *tkv^7^/+*; *cyfip^85.1^* (F), and *dad^J1E4^/+*; *cyfip^85.1^*/+ (G). Scale bar, 10 µm. (H) Statistical results for the number of satellite boutons from different genotypes. *n*≥15 for each genotype. * *p*<0.05, ** *p*<0.01, and *** *p*<0.001. Error bars indicate SEM.

Activation of BMP receptors upon ligand binding leads to the phosphorylation of the receptor-regulated R-Smad mothers against Dpp (Mad), which in turn activates the transcription of downstream genes. Therefore, the level of phosphorylated Mad (pMad) serves as a readout of BMP signaling at synaptic terminals. To test if BMP signaling was increased in *cyfip* mutants, we first quantified the pMad level at NMJ synapses by fluorescence immunohistochemistry and found that it was indeed significantly higher in *cyfip^85.1^* homozygous and hemizygous mutants than in wild type (*p*<0.001; Figure 5A–5C). We then examined the genetic interaction between *cyfip* and the components of the BMP signaling pathway. Mutating one copy of *mad* or *tkv*, which encodes the type I BMP receptor thickveins, significantly suppressed satellite bouton formation in *cyfip^85.1^* mutants (*p*<0.05 for both *mad^k00237^*/+; *cyfip^85.1^* and *tkv^7^/+*; *cyfip^85.1^* lines compared to *cyfip^85.1^* mutants; Figure 5D–5F and 5H). On the other hand, trans-heterozygotes of *cyfip^85.1^* and *dad^J1E4^* showed significantly more satellite boutons compared to wild type, while *cyfip^85.1^* or *dad^J1E4^* heterozygotes showed normal NMJ morphology (Figure 5G and 5H). *dad* encodes an inhibitory Smad that negatively regulates BMP signaling at the *Drosophila* NMJ synapses [32]. Together, these results indicate that the excess satellite bouton formation in *cyfip* mutants is caused by upregulated BMP signaling.

### Disruption of the presynaptic F-actin cytoskeleton in *cyfip* mutants

**Figure 6:**
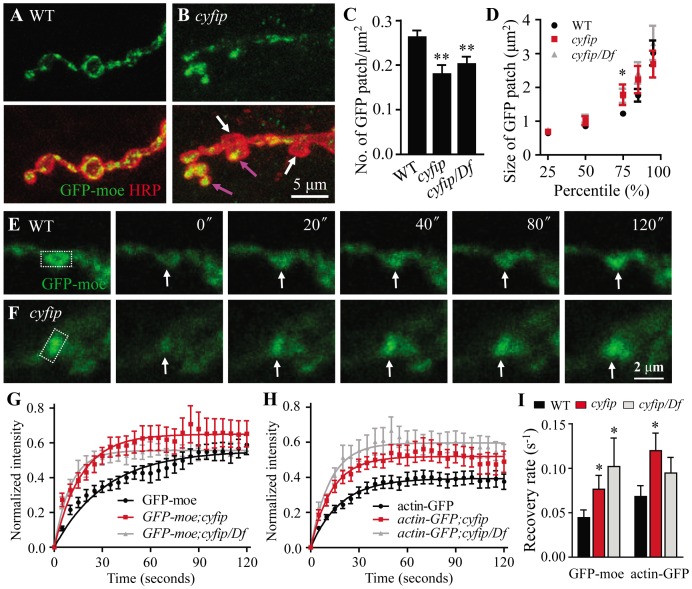
FRAP analysis shows increased F-actin formation at the NMJ terminals of *cyfip* mutants. (A, B) Presynaptically expressed GFP-moe driven by *elav-Gal4* revealed an uneven distribution of F-actin across different boutons in *cyfip^85.1^* mutants (compare boutons indicated by white and pink arrows in B). Scale bar, 5 µm. (C, D) Decreased total number but increased size of a sub-population at the 75^th^ percentile of GFP-moe patches in *cyfip^85.1^* mutant NMJs. *n* = 406 for the control; *n* = 115 for *cyfip* mutants; *n* = 211 for *cyfip/Df* mutants; * *p*<0.05; ** *p*<0.01. (E, F) Time-lapse images of GFP-moe expressed in the presynaptic terminals by *elav-Gal4* in wild type (E) and *cyfip^85.1^* mutants (F). The rectangular box indicates the region of interest (ROI) for photobleaching. Arrows indicate the position of recovered GFP-moe signals. Scale bar, 2 µm. (G, H) Relative GFP fluorescence intensities within the ROI after photobleaching of GFP-moe (G) and actin-GFP (H) patches. (I) Statistical results of fluorescence recovery rates (s^−1^) after photobleaching of presynaptically expressed GFP-moe and actin-GFP. *n*≥17 from 4 animals. Error bars indicate SEM.

Cyfip is a component of the heteropentameric WAVE complex that relays Rac1 signaling to the Arp2/3 nucleating complex to promote de novo actin polymerization. We therefore asked whether the synaptic phenotypes described above might result from a defect in the actin cytoskeleton. As the endogenous presynaptic actin cytoskeleton cannot be visualized by immunostaining, we expressed the GFP-moe reporter in motoneurons using *elav-Gal4*. A transgenic line expressing GFP fused to the C-terminal actin-binding domain of moesin (GFP-moe) has been used widely as an actin reporter [6], [14], [33], [34]. As shown in Figure 6A, when overexpressed in presynaptic neurons, GFP-moe concentrated into discrete puncta that distributed throughout wild-type NMJ terminals. These patches consist of F-actin, as they are stained by phalloidin and diffused after treatment with the F-actin disrupting drug latrunculin A [9], [14].

In *cyfip^85.1^* mutants, however, the presynaptic distribution of GFP-moe differed markedly between boutons; in some boutons, GFP-moe patches were large and spread throughout the whole bouton (pink arrow in Figure 6B), whereas in other boutons, no GFP-moe patches were observed (white arrow in Figure 6B). Quantitative analysis showed that the number of GFP-moe patches normalized to the synaptic area was significantly reduced in *cyfip* mutants (*p*<0.01; Figure 6C). The size distribution of F-actin patches was comparable between *cyfip* mutants and the control except that the patch size at the 75^th^ percentile was significantly increased from 1.22±0.08 µm^2^ in wild type to 1.78±0.30 µm^2^ and 1.74±0.21 µm^2^ in *cyfip^85.1^* homozygous and hemizygous mutants, respectively (*p*<0.05; Figure 6D). The uneven distribution of GFP-moe patches in different boutons of *cyfip^85.1^* mutants was also observed when an actin-GFP transgene was used as an actin marker (data not shown), confirming the abnormal presynaptic F-actin distribution in *cyfip* mutant NMJ synapses.

### Increased F-actin formation at the NMJ terminals of *cyfip* mutants

To evaluate F-actin dynamics, we performed fluorescent recovery after photobleaching (FRAP) assay in living synapses of GFP-moe labeled F-actin. GFP-moe was ectopically expressed in motoneurons driven by *elav-Gal4*. The region of interest (ROI, rectangles in Figure 6E and 6F) labeled with strong GFP signals at NMJ4 was used for photobleaching. A significant difference in the recovery of GFP intensity was observed within the first 20 s after photobleaching. The recovery from photobleaching was accelerated in mutants; fluorescence intensity within the ROI at 20 s returned to only 35.1±3.8% of the pre-bleached level in wild type (Figure 6E and 6G) but to 43.1±4.6% (*cyfip*) and 46.6±6.5% (*cyfip/Df*) in mutants (*p*<0.05; Figure 6F and 6G). The GFP intensity recovery rate was increased from 0.044±0.0087 s^−1^ in the wild-type controls to 0.076±0.015 s^−1^ in *cyfip^85.1^* homozygotes and 0.10±0.048 s^−1^ in hemizygotes, respectively (*p*<0.05; Figure 6I). To confirm that this fast recovery in *cyfip* mutants is caused by the dynamics of endogenous actin, we repeated the analysis using actin-GFP, an independent actin reporter. Again, a faster fluorescence recovery was observed in *cyfip^85.1^* homozygous mutants (*p*<0.05; Figure 6I). Collectively, the increased GFP fluorescence recovery indicates accelerated F-actin dynamics in *cyfip* mutants.

### New F-actin assembly is enhanced in *cyfip* mutants

**Figure 7:**
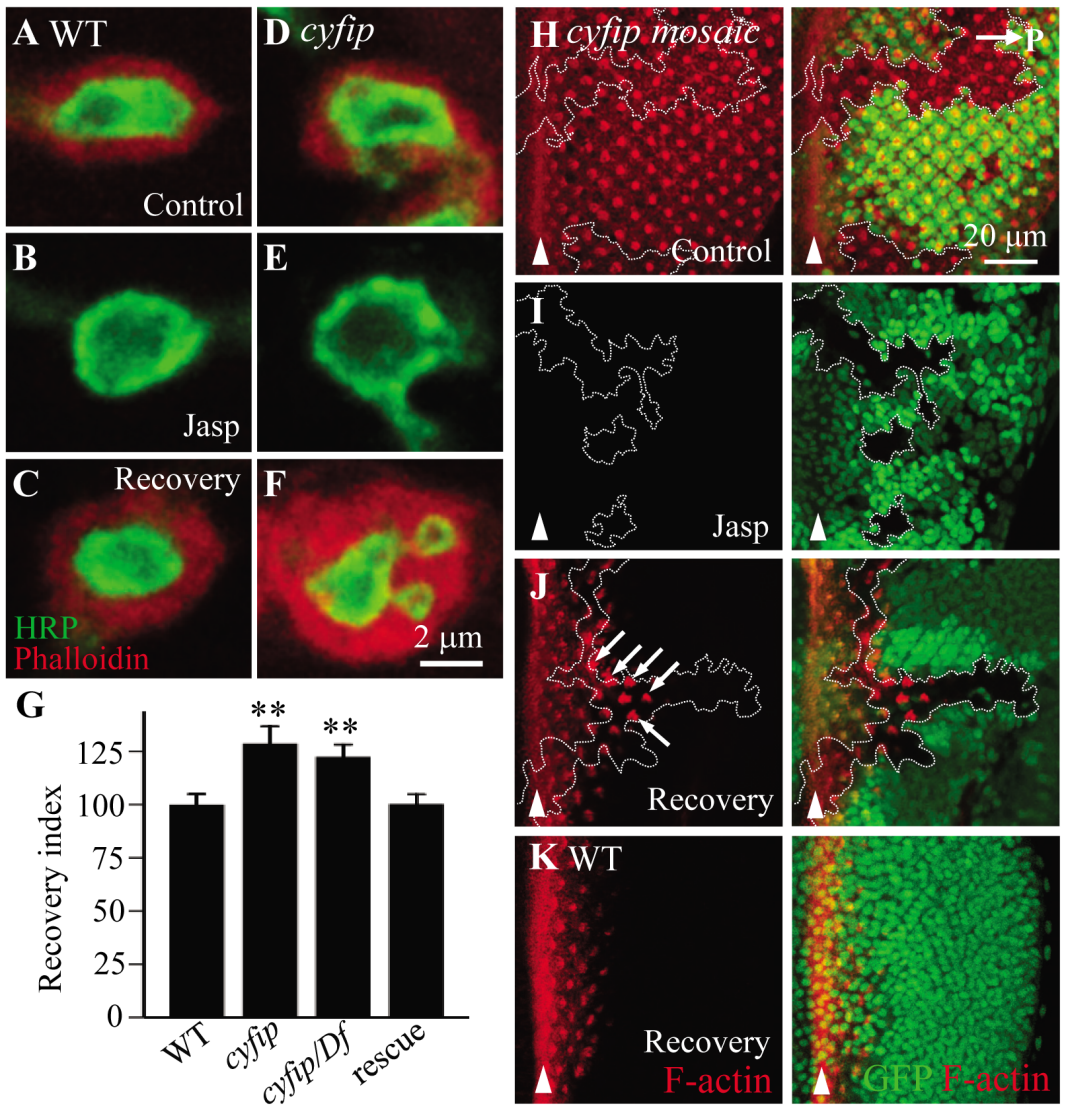
New F-actin assembly is enhanced in *cyfip* mutants. (A–F) Synaptic boutons of wild type (A–C) and *cyfip^85.1^* mutants (D–F) under different conditions: vehicle-treated control, treated with jasplakinolide, and after 1 h of jasplakinolide washout. The NMJ boutons were double-labeled with anti-HRP (green) and phalloidin (red) to reveals postsynaptic F-actin. (G) Fluorescence recovery index in the NMJ boutons of different genotypes after jasplakinolide treatment. At least 15 different boutons from 4 animals of each genotype were analyzed. (H–J) Phalloidin labeled (red) *cyfip* mosaic eye discs of third instar larvae under different conditions: vehicle-treated control (H), treated with jasplakinolide (I), and 90 min recovery after jasplakinolide washout (J). *cyfip^85.1^* mutant clones marked by the absence of GFP and outlined by white dotted lines show enhanced F-actin assembly in a subset of mutant cells (indicated by arrows). (K) F-actin recovery in a wild-type eye disc. Arrowheads indicate MF; horizontal arrow in H points to posterior.

To verify the accelerated actin dynamics in *cyfip* mutants, we applied a method developed to measure new F-actin assembly [35]. Endogenous F-actin at the presynaptic NMJ terminals is undetectable, but postsynaptic F-actin can be clearly visualized by phalloidin staining. The membrane-permeable drug jasplakinolide binds to and stabilizes pre-existing F-actin and prevents F-actin binding to phalloidin [36]. Thus, following jasplakinolide treatment, phalloidin will bind to only newly assembled F-actin. The distribution pattern and intensity of postsynaptic F-actin were similar in wild type and *cyfip^85.1^* mutants when treated with the vehicle DMSO (Figure 7A and 7D). After incubation with jasplakinolide for 15 min, postsynaptic F-actin was almost undetectable by phalloidin staining in both genotypes (Figure 7B and 7E). After 1 h recovery in jasplakinolide-free medium, phalloidin staining (selective for newly formed F-actin) was significantly higher in *cyfip^85.1^* mutants than in wild type (Figure 7C and 7F). We quantified newly formed F-actin by the fluorescence recovery index, defined as the ratio of phalloidin staining intensity at 1 h after jasplakinolide washout over the phalloidin staining intensity after vehicle-treatment. The recovery index indicated a significant increase in F-actin assembly in *cyfip^85.1^* homozygous and hemizygous mutants (*p*<0.01), which was rescued by ubiquitous expression of Cyfip driven by *act-Gal4* (Figure 7G). After 90 min recovery from jasplakinolide treatment, however, the F-actin intensity in homozygous *cyfip^85.1^* mutant NMJ terminals was comparable to the wild-type control (*n*≥18; *p*>0.05), suggesting that the increased F-actin formation in *cyfip* mutants is transient.

To further evaluate the role for Cyfip in new F-actin formation, we analyzed *cyfip* mosaic eye discs after drug treatment as described in a recent paper [37]. Development of *Drosophila* eye begins in the morphogenetic furrow (MF), where new ommatidia are formed as cells aggregate in periodic groupings. The new rows of ommatidial clusters are added anterior to older ones; consequently, the MF sweeps across the eye disc in a posterior to anterior direction [38]. In the region posterior to the MF, F-actin concentrates in the center of ommatidial clusters which will develop into rhabdomeres (Figure 7H). When the eye discs were treated with the vehicle DMSO, we observed similar intensity and distribution pattern of F-actin in *cyfip* mutant clones compared with the adjacent wild-type tissue (Figure 7H), consistent with the results observed at the postsynapse (Figure 7A and 7D). After incubation of jasplakinolide for 10 min, phalloidin staining detected no signal in the whole disc as the existing F-actin was totally masked by jasplakinolide binding (Figure 7I). Following 90 min recovery in jasplakinolide-free medium, F-actin started to form in the region posterior to the MF (Figure 7K). In *cyfip^85.1^* mutant clones, we found enhanced and advanced F-actin recovery in a subpopulation of *cyfip* mutant cells, presumably undergoing active morphogenesis, compared to the nearby wild-type tissue (arrows in Figure 7J). As the recovery continues, additional phalloidin labeling could be detected in the more posterior ommatidial clusters. These results together show elevated F-actin formation within the examined time window in *cyfip* mutants.

### Cyfip functionally antagonizes SCAR

Cyfip is a component of the WAVE complex. In vitro biochemical studies show that in the resting state, the WAVE complex is inactive. Upon binding of Rac1 to the N-terminus of Cyfip, the VCA domain of WAVE protein is released from the complex to activate F-actin assembly through the actin nucleator Arp2/3 [18], [21]–[23]. However, it is unknown if Cyfip regulates WAVE activity and subsequent F-actin assembly in vivo through the same mechanism.

**Figure 8:**
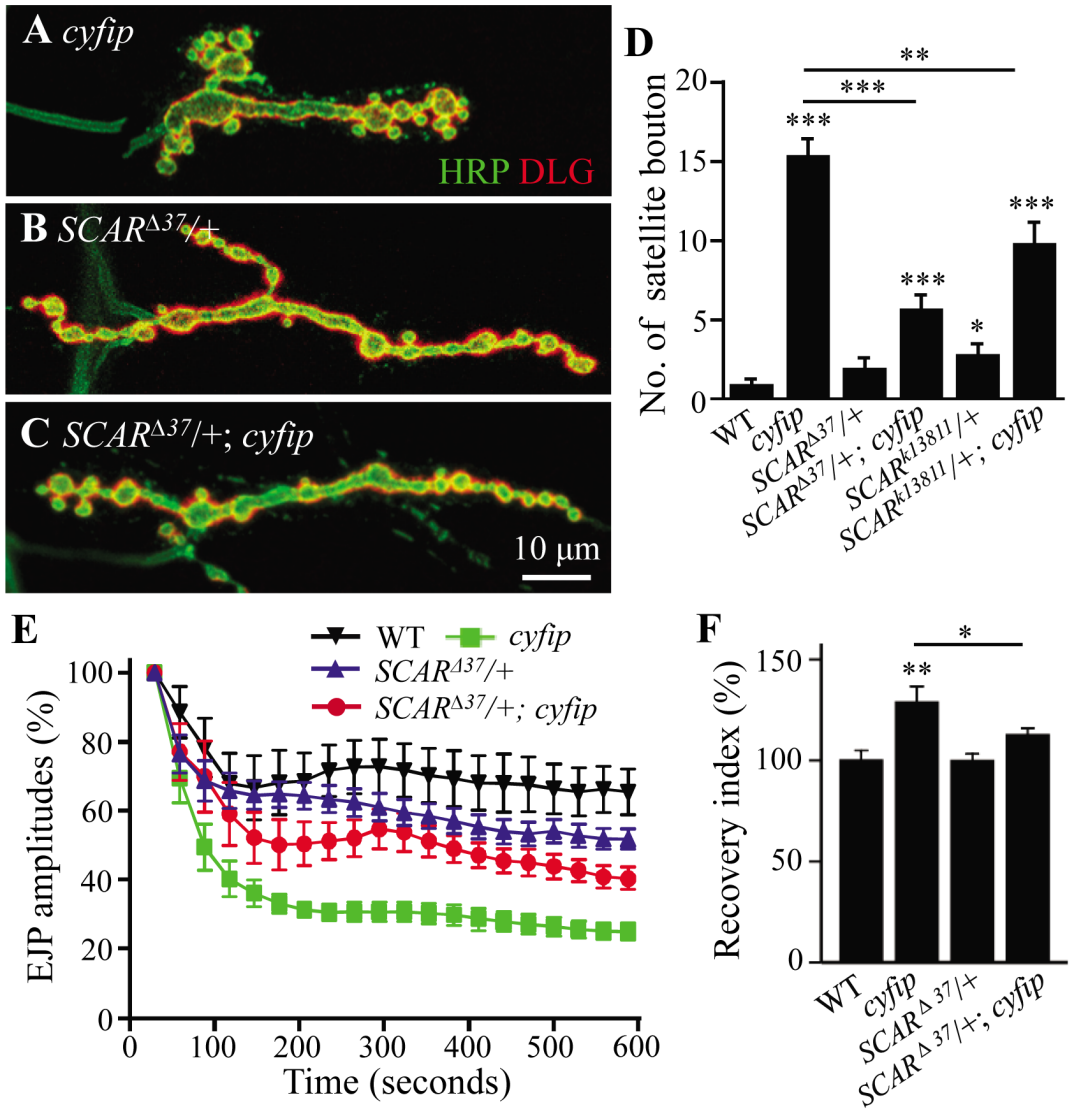
Cyfip functionally antagonizes SCAR in multiple contexts. (A–C) Representative NMJ terminals of different genotypes. Scale bar, 10 µm. (D) Statistical analysis of the number of satellite boutons in different genotypes. The heterozygous *SCAR* mutant alleles *SCAR^Δ37^* or *SCAR^k13811^* rescued the excessive satellite bouton phenotype of *cyfip^85.1^* mutants. (E) Mutating a copy of *SCAR* rescued the enhanced decline of EJP amplitudes during 10 Hz stimulation in *cyfip^85.1^* mutants. (F) Mutating a copy of *SCAR* restored the increased F-actin assembly rate in *cyfip^85.1^* mutants to the wild-type level. *n*≥8 for each genotype; * *p*<0.05, ** *p*<0.01, and *** *p*<0.001; error bars indicate SEM.

Enhanced F-actin assembly (Figure 6 and Figure 7) suggests an increased activity of SCAR, the *Drosophila* homolog of WAVE, in *cyfip* mutants. We demonstrated that this was indeed the case by examining NMJ morphology and synaptic transmission in *cyfip^85.1^* null mutants with a copy of *SCAR* mutated. Heterozygous *SCAR^Δ37^* (a null allele) or *SCAR^k13811^* (a hypomorphic allele) mutations reversed the increased number of satellite boutons in *cyfip^85.1^* mutants (*p*<0.001 for *SCAR^Δ37^*/+; *cyfip^85.1^* and *p*<0.01 for *SCAR^k138117^*/+; *cyfip^85.1^* compared to *cyfip^85.1^* mutants; Figure 8A–8D). Similarly, the enhanced rundown of EJP amplitude under tetanic stimulation was also partially restored from 24.8±2.4% to 40.3±2.9% of the initial EJP amplitude when a copy of *SCAR* was mutated in the *cyfip* null background (*SCAR^Δ37^*/+; *cyfip^85.1^*) (Figure 8E). Reducing the dose of *SCAR* also rescued the accelerated F-actin assembly at the postsynaptic site of *cyfip^85.1^* mutants. The fluorescence recovery index was 112.52±3.49% in *SCAR^Δ37^*/+; *cyfip^85.1^* mutants, significantly lower than 128.72±7.93% in *cyfip^85.1^* mutants (*p*<0.05; Figure 8F). As a control, heterozygous *SCAR^Δ37^* or *SCAR^k13811^* mutants showed no obvious defects in synaptic morphology, endocytosis or F-actin assembly (Figure 8D–8F). Together, these data indicate that the synaptic defects and accelerated F-actin assembly in *cyfip^85.1^* mutants result at least partially from increased SCAR activity.

## Discussion

Supernumerary buds, i.e., excess satellite boutons at NMJs are observed in both *cyfip* null mutants and animals with *cyfip* knocked down specifically in presynaptic neurons by RNA interference [24], [39]. However, little is known of how Cyfip regulates synapse development. Here, we provide experimental evidence for a model in which Cyfip regulates the development and endocytosis of NMJ synapses by inhibiting F-actin assembly.

### Cyfip suppresses F-actin assembly by antagonizing WAVE

The Arp2/3 complex, which is activated by the WASP family proteins including WASP and WAVE, mediates nucleation of de novo F-actin assembly. WASP is auto-inhibited, whereas the activity of the WAVE protein is inhibited in the heteropentameric WAVE complex. Two models have been proposed to explain WAVE complex activation in vitro. One model proposes that Rac1 binding to Cyfip causes dissociation of the WAVE complex, releasing the active WAVE-containing subcomplex and resulting in actin nucleation by Arp2/3 [19]. The other model hypothesizes that upon binding of Rac1 to Cyfip, the WAVE complex is activated through an allosteric change rather than by dissociation of the complex to expose the VCA domain [21], [23]. However, the mechanisms regulating the activity of the WAVE complex in vivo remain unclear.

Like its mammalian counterpart, *Drosophila* Cyfip is a component of the heteropentameric WAVE complex as evidenced by co-immunoprecipitation assays and the strong mutual dependence on the protein levels of the individual components [40], [41], which we confirmed (Figure S2). If the integrity of the WAVE complex is necessary for its activity, then loss of Cyfip would disrupt the complex, leading to reduced Arp2/3 activity and slower F-actin assembly. However, we showed that loss of Cyfip accelerated F-actin formation in vivo. First, FRAP analysis at NMJ synapses revealed a faster recovery of GFP-moe fluorescence in *cyfip* mutants than in wild type (Figure 6). Second, new F-actin assembly was accelerated at NMJ terminals and eye discs of *cyfip* mutants (Figure 7). Third, genetic analysis showed that mutating a copy of SCAR reversed the *cyfip* mutant phenotypes including excessive satellite bouton formation and accelerated F-actin assembly (Figure 8). The antagonistic interaction between *cyfip* and *SCAR* resembles that between *SCAR* and *kette*, the *Drosophila* homolog of *Nap1*. *Drosophila kette* mutants show fused commissures in the embryonic nervous system that are rescued by reducing the dose of *SCAR* [42]. Together, in vivo studies from independent assays reveal an upregulation of SCAR activity when the WAVE/SCAR complex is disrupted in *cyfip* mutants. We envision that in the absence of Cyfip, the WAVE complex is dissociated, leaving the VCA domain exposed to activate Arp2/3 and promote F-actin assembly.

Klambt and colleagues reported a role for Cyfip in F-actin formation [39]. When *cyfip* is knocked down by RNAi in S2 cells, there is an accumulation of cytosolic F-actin; when the expression of Cyfip is up- or downregulated, F-actin formation during bristle development is altered; they concluded that Cyfip affects F-actin formation though the specific mechanism was unclear [39]. In agreement with their findings, our results from phalloidin staining of steady-state samples did not show consistent alterations in the level of F-actin in *cyfip* mutants (i.e. normal F-actin at the postsynapse, abnormal ring canal and radial F-actin fibers in nurse cells, but absence of F-actin in the cortex of nurse cells; Figure 7 and Figure S3), suggesting that Cyfip-regulated F-actin formation is dependent on cellular contexts. However, our analyses of live imaging and pharmacological treatment independently showed increased new F-actin assembly, though the steady-state F-actin level was normal at NMJ terminals and eye discs of *cyfip* mutants (Figure 6 and Figure 7), suggesting that the increased F-actin assembly in *cyfip* mutants could be temporary. We suppose that a complex regulatory network is at work to maintain the dynamics of F-actin. Lack of *cyfip* impairs the “brake” and results in inappropriate or ectopic F-actin polymerization. However, enhanced formation of F-actin in *cyfip* mutants could be quickly mitigated by proteasomal degradation of SCAR ([40] and Figure S2), activation of depolymerizing factors, or both.

In addition to being a component of the WAVE complex that regulates actin dynamics, Cyfip also interacts with the translational regulator FMRP and the translation initiation factor eIF4E [24], [43]. It is possible that Cyfip interacts with different partners to mediate different cellular processes. We note that while a synaptic role of eIF4E has not been demonstrated, *cyfip* and *dfmr1* mutants show distinct NMJ phenotypes [24], [44]. How Cyfip coordinates with these different partners to regulate synapse formation and function remains to be elucidated.

### Cyfip plays a regulatory role in synaptic endocytosis

Multiple lines of evidence suggest a role for Cyfip in endocytosis at *Drosophila* NMJ synapses. First, *cyfip* mutant NMJ synapses exhibited prominent satellite boutons, a phenotype well documented in several endocytic mutants [15], [16], [26], [27], [29]. Second, genetic interaction analysis showed a synergistic effect between *cyfip* and endocytic genes such as *dynamin*, *dap160*, and *endophilin* in the control of satellite bouton formation (Figure S4). Third, EM analysis revealed enlarged SVs at active zones and a significant increase in the number of cisternae in *cyfip* mutant NMJ boutons (Figure 2). Consistent with enlarged SVs, there was a higher frequency of large mEJPs in *cyfip* mutants (Figure 3). Last, *cyfip* mutant synapses could not sustain neurotransmission during high-frequency stimulation and displayed a slower recovery of neurotransmission following tetanic stimulation (Figure 4). We also performed FM1–43 dye loading assay and found normal dye uptake at *cyfip* mutant NMJs (data not shown). FM dye loading is commonly used for examining endocytosis. However, there are reports documenting that endocytic mutants showed normal FM dye uptake. For example, clathrin is a critical component of endocytic machinery, but acute inactivation of clathrin showed normal FM loading [45]. These results together support a role for Cyfip in synaptic endocytosis.

The actin cytoskeleton has been implicated in multiple steps of the endocytic pathway, from membrane invagination to vesicle fission and subsequent trafficking [46]–[48]. At which step of endocytosis might Cyfip act? Synaptic vesicles are generated either directly from plasma membrane through clathrin-mediated endocytosis or from endosomal compartments derived from bulk endocytosis during intense stimulation. Electron microscopic analysis revealed normal SV density in *cyfip* mutants, indicating that membrane retrieval capacity is not altered by loss of Cyfip (Figure 2). Consistently, the FM1–43 dye uptake assay showed largely normal endocytosis and vesicle pool in *cyfip* mutants (data not shown). However, *cyfip* mutant NMJ boutons show increased vesicle size, which is tightly controlled by clathrin and its adaptor proteins during endocytosis [30], [45]. It is well documented that endocytic mutants of *Dap160/intersectin* and *Eps15* exhibit enlarged vesicle size and these endocytic proteins interact with actin regulators Nwk and WASP [7], [15], [16], [27]. Thus, Cyfip-regulated actin cytoskeleton may affect SV size through endocytosis directly or indirectly.

### Cyfip inhibits satellite bouton formation

We report here that Cyfip normally suppresses F-actin assembly to restrain satellite bouton formation. We showed that F-actin distributed unevenly among different boutons of *cyfip* mutants (Figure 6). The F-actin cytoskeleton was also disrupted in egg chambers (Figure S3). Furthermore, we found upregulated F-actin assembly in *cyfip* mutants, which might cause the mislocalization of F-actin filaments in neuronal and non-neuronal cells (Figure 6, Figure 7, and Figure S3). Both the enhanced actin dynamics and aberrant NMJ morphology in *cyfip* mutants were rescued by reducing the dose of *SCAR*. Based on these findings, we propose that Cyfip normally restrains satellite bouton formation by suppressing F-actin assembly through the SCAR-Arp2/3 pathway.

It is well established that an actin-dependent endocytic mechanism contributes to the formation of satellite boutons. Loss of Nwk, an SH3 adaptor protein that interacts with Cdc42 and WASP, also impairs endocytic attenuation of BMP signaling and results in excess satellite bouton formation [7], [8], but SV endocytosis was normal in *nwk* mutants as evidenced by FM1–43 dye uptake and EJP recordings under high-frequency stimulation [25]. Here, we report that, like *nwk* mutants, the excess satellite boutons in *cyfip* mutants are also due to upregulated BMP signaling (Figure 5). In contrast to *nwk* null mutants, however, *cyfip* mutants showed defective endocytosis as revealed by EM and electrophysiological analysis (Figure 2, Figure 3, Figure 4). Thus, *nwk* and *cyfip* may regulate synaptic endocytosis through distinct actin-mediated pathways; *nwk* primarily affects endocytic regulation of BMP signaling, while *cyfip* regulates endocytosis of both SVs and BMP receptors. In any case, mutations in both genes lead to formation of excess satellite boutons as a result of dysregulated actin dynamics.

## Materials and Methods

### *Drosophila* strains and genetics

Fly cultures were raised on conventional cornmeal medium and maintained at 25°C unless specified. *w^1118^* flies were used as the wild-type controls in all experiments. *cyfip^85.1^* and *UAS*-*cyfip* were gifts of Dr. A. Giangrande [24]. *Df(3R)Exel6174* (88F1–88F7) covering the *cyfip* locus (88F1) was obtained from the Bloomington Stock Center. The BMP pathway mutants *mad^k00237^*, *tkv^7^*, and *dad^J1E4^*, and the *SCAR* mutants *SCAR^Δ37^* (from the Bloomington Stock Center) and *SCAR^K13811^* (from the Kyoto *Drosophila* Genetic Resource Center) were used for genetic interaction analysis. Transgenic flies carrying *UAS-GFP-moesin* were from S. Hayashi [33]. A line carrying *UAS-actin-GFP* was from the Kyoto *Drosophila* Genetic Resource Center. For overexpression or rescue experiments, the pan-neuronal *elav-Gal4*, the muscle-specific *C57-Gal4*, and the ubiquitously expressed *act-Gal4* drivers were used. *hs-FLP*; *FRT82B cyfip^85.1^* females were crossed to *FRT82B ubi-GFP* males to generate mosaic clones in eye discs [37].

### Immuno-histochemical analysis

For immunostaining of NMJ synapses, wandering third-instar larvae were dissected in Ca^2+^-free standard saline, and then fixed in fresh 4% paraformaldehyde for 30 min. The monoclonal mouse antibody anti-DLG (4F3; 1∶1000) was from the Developmental Studies Hybridoma Bank at the University of Iowa. FITC-conjugated goat anti-HRP and Alexa 568-conjugated goat anti-HRP (1∶100 for both) were from Jackson ImmunoResearch. Texas-red phalloidin (1∶6) was from Molecular Probes. The secondary antibodies used were Alexa 488-, or 568-conjugated anti-mouse or anti-rabbit from Invitrogen (1∶1000). F-actin in eye discs was labeled with Texas-red phalloidin at 1∶400. All images were collected using a Leica SP5 laser scanning confocal microscope and processed with Adobe Photoshop 8.0.

For statistical analysis, type Ib terminals of NMJ4 in abdominal segment A2 or A3 were quantified. A donut-shaped anti-DLG staining pattern indicated a single bouton. Satellite boutons were defined as small boutons budding from major synaptic terminals or primary boutons. Superboutons were defined as the parental boutons around which three or more satellite boutons formed. The superboutons were categorized into three groups bearing 3, 4, or ≥5 satellite boutons. The length of the NMJ was measured by Image J based on HRP-stained terminals. For presynaptic GFP-moe patch analysis, we quantified the number and area of GFP patches over 0.5 µm^2^ by ImageJ. The number of GFP patches was normalized to the synaptic areas delineated by anti-HRP staining.

### Electron microscopy

Larval neuromusculature was prepared for EM analysis according to procedures described previously [49]. Dissected larvae were fixed for 2 h with 2.5% glutaraldehyde (Sigma-Aldrich) in cacodylate buffer (pH 7.4) at room temperature followed by several rinses with cacodylate buffer. Right and left hemi-segments from abdominal segments A3 and A4 were separated from the larval fillets and post-fixed with 1% OsO_4_ in cacodylate buffer for 2 h. The preparations were stained en bloc for 1 h with saturated uranyl acetate in 50% ethanol before dehydration in an ethanol series. The samples were embedded in Spurr resin (Sigma). Longitudinal ultra-thin sections were made on an LKB ultra-microtome or Leica UC6 ultra-microtome using a diamond knife. Grids were post-stained with saturated uranyl acetate in 50% ethanol and 1% lead citrate (pH 12) and examined under a JEOL 1010 electron microscope. Electron micrographs were acquired using a Ganton792 digital CCD. For quantification, we analyzed cross-sections through the midline of more than 20 individual boutons from 5 animals of each genotype. The number and diameter of synaptic vesicles within a 200 nm radius of the active zone were measured by ImageJ. Vesicle structures with diameters >60 nm were defined as cisternae.

### Electrophysiological assays

Intracellular recordings were performed at 20°C following published procedures [17], [49] with minor modifications. Briefly, to assess basal neuromuscular transmission, wandering third instar larvae were dissected in modified HL3 saline (NaCl 70 mM, KCl 5 mM, MgCl_2_ 10 mM, NaHCO_3_ 10 mM, sucrose 115 mM, D-trehalose 5 mM, and HEPES 5 mM, pH 7.2) and evoked responses recorded in modified HL3 saline containing 0.23 mM Ca^2+^. Intracellular microelectrodes with a resistance of 8–20 MΩ when filled with 3 M KCl were used for recording. Recordings were performed using an Axoclamp 2B amplifier (Axon Instruments) in Bridge mode. Data were filtered at 1 kHz and digitized using Digitizer 1322A (Axon Instruments). Data acquisition was controlled by Clampex 9.1 software. Excitatory junctional potentials (EJPs) were evoked at 0.3 Hz by a suction electrode using a depolarizing pulse delivered by a Grass S48 stimulator (Astro-Grass Inc.). Both EJPs and miniature EJPs (mEJPs) were recorded from muscle 6 of abdominal segment A2 or A3, and processed with Clampfit 10.2 software. Quantal content was calculated by dividing the EJP amplitude (after correction for nonlinear summation) by the mEJP amplitude according to a classic protocol [50]. The EJP correction for nonlinear summation assumed a reversal potential of 10 mV. To examine synaptic transmission under high frequency stimulation, synapses were stimulated at 10 Hz for 10 min and evoked EJPs recorded in modified HL3 saline with 10 mM Ca^2+^. Immediately following the high-frequency train, EJPs in response to 0.3 Hz stimulation were recorded for 10 min. The amplitudes of EJPs within successive 30 s intervals were averaged and normalized to the initial amplitude to yield the time course of recovery. At least 10 NMJ preparations were recorded for each genotype.

### FRAP analysis

For fluorescence recovery after photobleaching (FRAP) analysis, actin-GFP or GFP-moe was expressed presynaptically by *elav-GAL4*. Third instar larvae were dissected in modified HL3 saline (NaCl 70 mM, KCl 5 mM, MgCl_2_ 10 mM, NaHCO_3_ 10 mM, sucrose 115 mM, D-trehalose 5 mM, and HEPES 5 mM, pH 7.2) and imaged using a 40× water immersion lens on a Leica SP5 confocal microscope. The GFP fluorescence at NMJ synapses was bleached by scanning the region of interest (ROI, 1×2 µm) using full power of the 488 nm Argon laser line for 8 s with a pinhole setting of 2.0 airy units. The images were captured at zoom 8 with a resolution of 512×512 pixels. Recovery images were collected with low-intensity 488 nm excitation light to avoid additional bleaching. The fluorescence intensity within the ROI was calculated using ImageJ software. The relative fluorescence emission intensity F(t) was calculated as follows: F(t) = (Ft–Fb)/(Fi–Fb)×100%, where Ft is the fluorescence intensity within the ROI at some time (t) after bleaching, Fb is the background intensity, and Fi is the initial fluorescence intensity before bleaching [51]. The recovery data points were fitted to a one-phase exponential equation. The recovery rate k was calculated from k = 0.693/t_1/2_ [52], in which t_1/2_ represents half time, the time for the recovered GFP to reach 50% of the fluorescence intensity plateau. At least 17 serial recordings from 4 animals were used for statistical analysis.

### Examination of new F-actin assembly

New F-actin assembly was examined as previously described [35] with minor modifications. Briefly, jasplakinolide (Invitrogen) was dissolved in DMSO to make a 1 mM stock solution. To visualize newly polymerized F-actin, dissected samples were pretreated with jasplakinolide at a concentration of 10 µM for 15 min for NMJ synapses or 10 min for eye discs. After recovery in the drug-free medium (60 min for NMJ synapses, 90 min for eye discs), preparations were fixed and stained with Alexa-546-conjugated phalloidin (1∶400, Invitrogen) to detect newly-polymerized F-actin. Controls were treated with the vehicle DMSO only. For quantitative analysis of fluorescence intensity, samples were processed simultaneously and imaged using identical acquisition parameters. Serial images of eye discs and 1 µm single section of NMJ4 boutons in abdominal segments A2 or A3 were collected using a Leica SP5 laser scanning confocal microscope. The intensity of phalloidin staining in individual boutons was analyzed using ImageJ software (NIH). The recovery index RI was calculated as follows: RI = (I_re_–I_jasp_)/I_ctl_×100%, where I_re_ is the phalloidin intensity after 1 h recovery, I_jasp_ is the phalloidin intensity immediately after jasplakinolide treatment, and I_ctl_ is the phalloidin intensity with vehicle treatment. Statistical analysis was performed using ANOVA for comparison of multiple group means.

## Supporting Information

Figure S1 Satellite boutons form primarily in late larval stages in *cyfip* mutants. (A–H) Representative NMJ4 synapses from different larval stages, early 2nd instar (e-2nd), late 2nd instar (l–2nd), early 3rd instar (e-3rd), middle 3rd instar (m-3rd), and wandering 3rd instar (w-3rd) larvae, were double-stained with anti-HRP (green) and anti-DLG (red). Wild-type synapses grew continuously with increased NMJ length (A–D). A synaptopod is indicated by an arrow in (A). N indicates nerves. (E–H), *cyfip8^5.1^* null mutant synapses grew slower from e-2nd instar with shorter NMJ length. Synaptopods are indicated by arrows in (E) and (F); satellite boutons are denoted by asterisks in (G) and (H). Scale bar, 10 µm. (I) The correlation between the NMJ length and muscle surface area in wild type and *cyfip^85.1^* mutants. The developmental curves were fit by a power regression model (*r* = 0.81 for wild type and *r* = 0.66 for mutants). *n*≥50 for each genotype. (J) Quantification of satellite boutons at various larval stages in wild type and *cyfip* mutants. Satellite boutons in *cyfip^85.1^* null mutants appeared from early larval stages, and the number of satellite boutons increased sharply starting from m-3rd instar. *n*≥8 for each time point; error bars indicate SEM.
(TIF)

Figure S2 Loss of Cyfip results in a decreased level of SCAR at NMJ terminals. (A–B) Representative NMJ4 synapses from wild type (A) and *cyfip^85.1^* mutants (B) co-stained with anti-HRP (red) and anti-SCAR (green), a gift from Dr. Sven Bogdan (Bogdan et al., 2005). Lower left panel shows that SCAR is significantly reduced in the NMJ terminal of *cyfip* mutants.
(TIF)

Figure S3 Abnormal F-actin formation during oogenesis of *cyfip* mutants. (A–H) A stage 10A egg chamber was co-stained with Texas red-conjugated phalloidin (red) and anti-Hts (green), a marker for ring canals. (B–D) Enlarged views of the wild-type ring canal shown in (A). (F–H) Enlarged views of the mutant ring canal shown in (E). *cyfip^85.1^* germline clones were generated following a conventional protocol (Chou and Perrimon, 1996). nc denotes nurse cells; oo indicates oocytes. Scale bars in (A, E) and (B–D and F–H) represent 40 and 10 µm, respectively. (I, J) Nurse cells of stage 10B egg chambers stained with Texas red-conjugated phalloidin from wild type (I) and *cyfip^85.1^* mutants (J). Arrows indicate cytoplasmic actin filaments, while white dots denote subcortical F-actin. Scale bar, 40 µm. (K, L) Nomarski images of stage 13 egg chambers from wild type (K) and *cyfip^85.1^* mutants (L) demonstrating a cytoplasmic dumping defect in nurse cells in the mutant egg chamber. Scale bar, 100 µm.
(TIF)

Figure S4 Satellite boutons in *cyfip* mutants are dominantly enhanced by endocytic mutants. (A–F) Endocytic mutants *shi^1^* (a temperature sensitive allele), *dap160^Δ1^* (a null or severe hypomorph), *endoA^Δ4^* (a null allele), and *endoA^EY02730^* (a hypomorph) (all from the Bloomington Stock Center) were used for genetic interaction analysis. Representative images of NMJ4 synapses labeled with anti-HRP from *cyfip^85.1^*/+ (A), *shi^1^*/+; *cyfip^85.1^*/+ (B), *dap160^Δ1^*/+; *cyfip^85.1^*/+ (C), *cyfip^85.1^* null mutants (D), *shi^1^*/+; *cyfip^85.1^* (E), and *dap160^Δ1^*/+; *cyfip^85.1^* (F). Scale bar, 10 µm. (G, H) Statistical results of the number of satellite boutons (G) and superboutons (H) from different genotypes at 25°C. *n*≥16 for each genotype. * *p*<0.05, ** *p*<0.01, and *** *p*<0.001; error bars indicate SEM.
(TIF)
