# Supplementary figures and images for: Drosophila cyfip Regulates Synaptic Development and Endocytosis by Suppressing Filamentous Actin Assembly
Source: PLoS Genet. 2013 Apr 4;9(4):e1003450. doi: 10.1371/journal.pgen.1003450 (PMC3616907; doi:10.1371/journal.pgen.1003450)

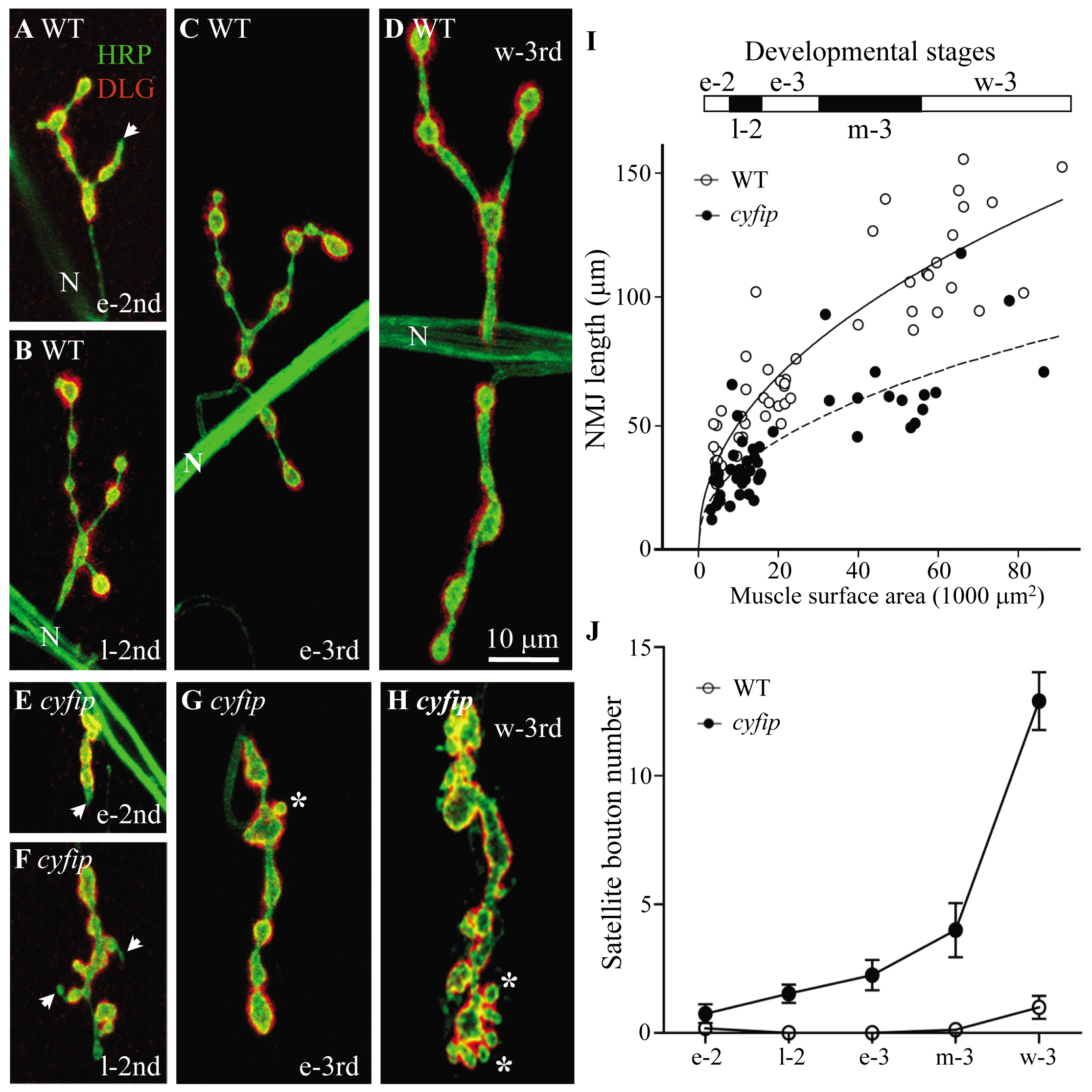

Supplement: Figure S1 — Satellite boutons form primarily in late larval stages in cyfip mutants. (A–H) Representative NMJ4 synapses from different larval stages, early 2nd instar (e-2nd), late 2nd instar (l–2nd), early 3rd instar (e-3rd), middle 3rd instar (m-3rd), and wandering 3rd instar (w-3rd) larvae, were double-stained with anti-HRP (green) and anti-DLG (red). Wild-type synapses grew continuously with increased NMJ length (A–D). A synaptopod is indicated by an arrow in (A). N indicates nerves. (E–H), cyfip85.1 null mutant synapses grew slower from e-2nd instar with shorter NMJ length. Synaptopods are indicated by arrows in (E) and (F); satellite boutons are denoted by asterisks in (G) and (H). Scale bar, 10 µm. (I) The correlation between the NMJ length and muscle surface area in wild type and cyfip85.1 mutants. The developmental curves were fit by a power regression model (r = 0.81 for wild type and r = 0.66 for mutants). n≥50 for each genotype. (J) Quantification of satellite boutons at various larval stages in wild type and cyfip mutants. Satellite boutons in cyfip85.1 null mutants appeared from early larval stages, and the number of satellite boutons increased sharply starting from m-3rd instar. n≥8 for each time point; error bars indicate SEM. (TIF) [file pgen.1003450.s001.tif]

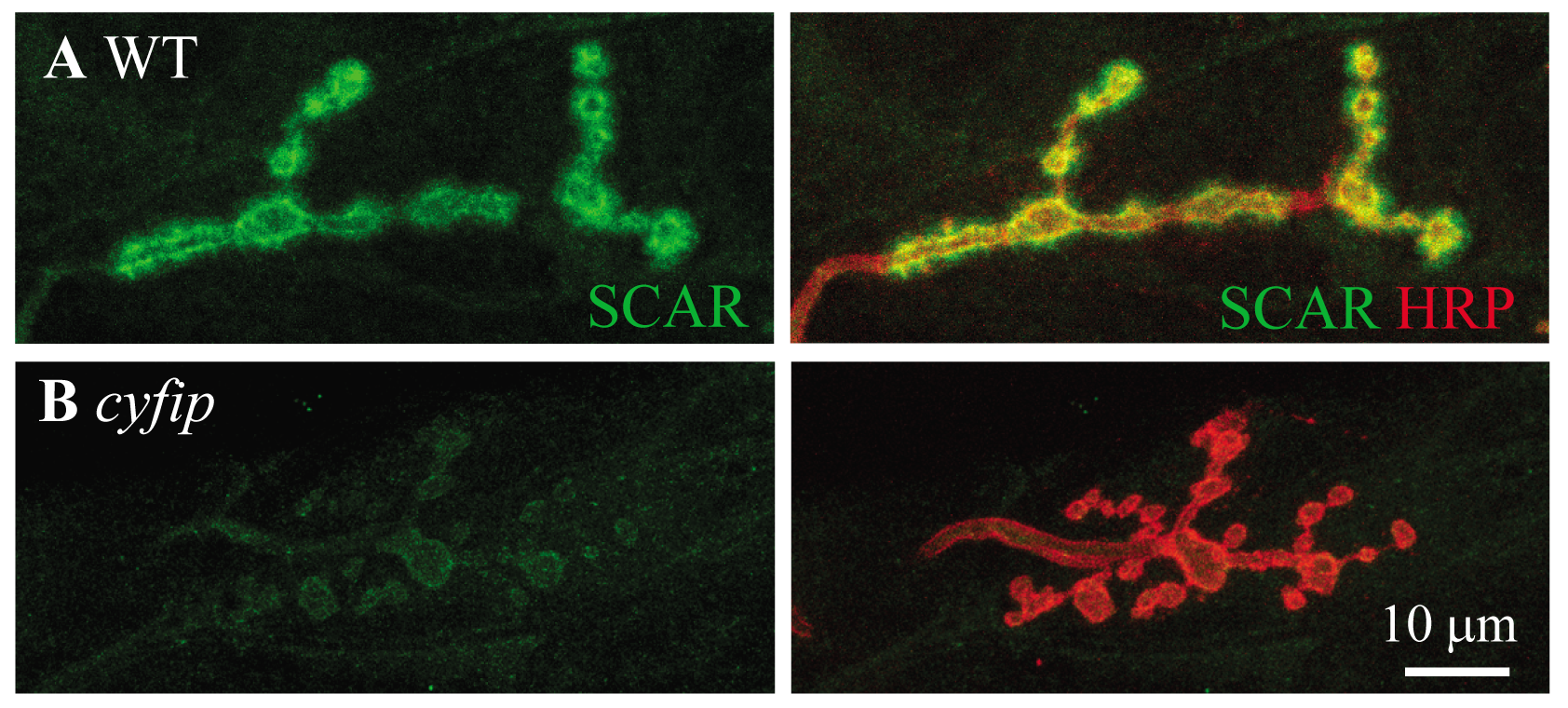

Supplement: Figure S2 — Loss of Cyfip results in a decreased level of SCAR at NMJ terminals. (A–B) Representative NMJ4 synapses from wild type (A) and cyfip85.1 mutants (B) co-stained with anti-HRP (red) and anti-SCAR (green), a gift from Dr. Sven Bogdan (Bogdan et al., 2005). Lower left panel shows that SCAR is significantly reduced in the NMJ terminal of cyfip mutants. (TIF) [file pgen.1003450.s002.tif]

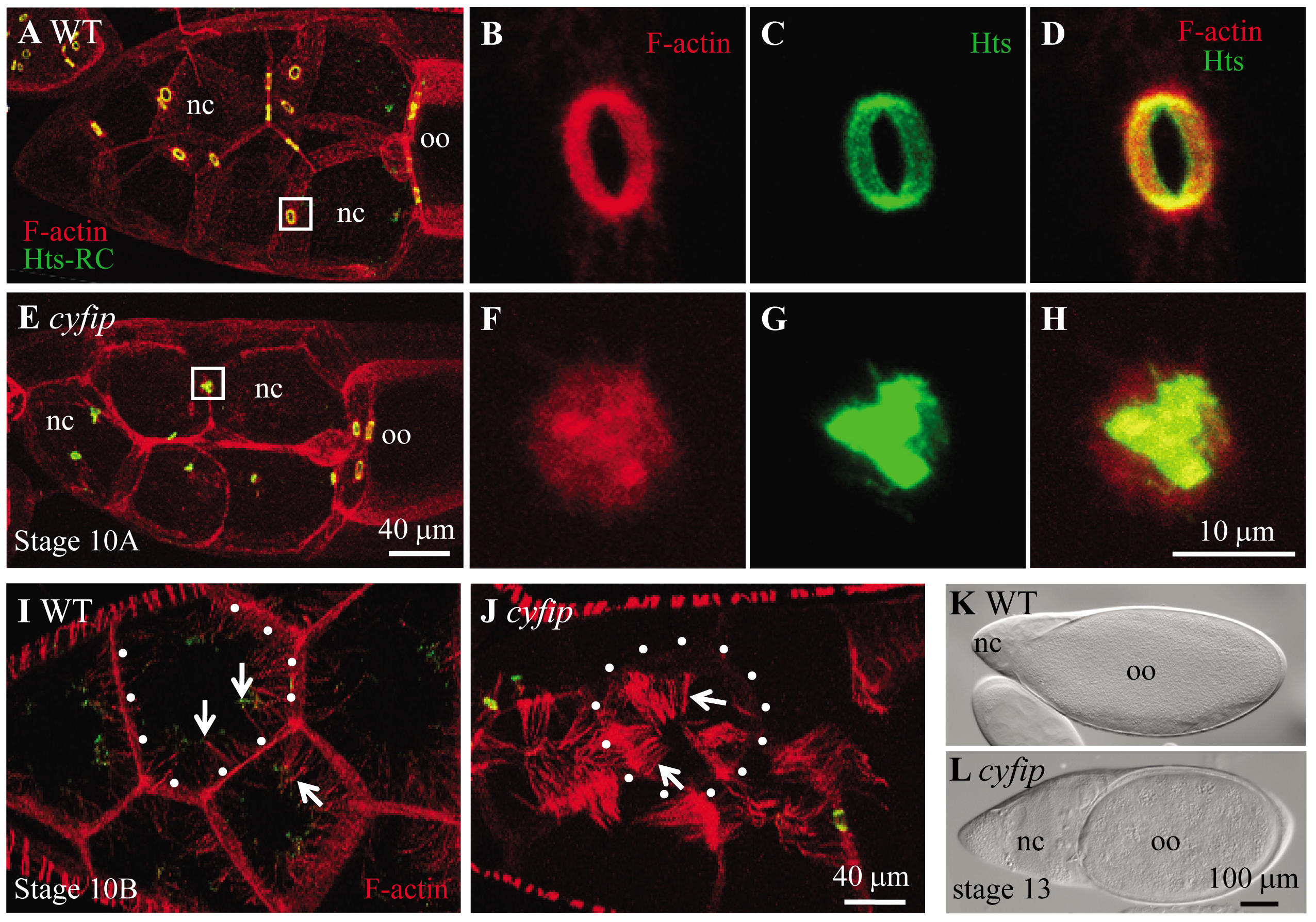

Supplement: Figure S3 — Abnormal F-actin formation during oogenesis of cyfip mutants. (A–H) A stage 10A egg chamber was co-stained with Texas red-conjugated phalloidin (red) and anti-Hts (green), a marker for ring canals. (B–D) Enlarged views of the wild-type ring canal shown in (A). (F–H) Enlarged views of the mutant ring canal shown in (E). cyfip85.1 germline clones were generated following a conventional protocol (Chou and Perrimon, 1996). nc denotes nurse cells; oo indicates oocytes. Scale bars in (A, E) and (B–D and F–H) represent 40 and 10 µm, respectively. (I, J) Nurse cells of stage 10B egg chambers stained with Texas red-conjugated phalloidin from wild type (I) and cyfip85.1 mutants (J). Arrows indicate cytoplasmic actin filaments, while white dots denote subcortical F-actin. Scale bar, 40 µm. (K, L) Nomarski images of stage 13 egg chambers from wild type (K) and cyfip85.1 mutants (L) demonstrating a cytoplasmic dumping defect in nurse cells in the mutant egg chamber. Scale bar, 100 µm. (TIF) [file pgen.1003450.s003.tif]

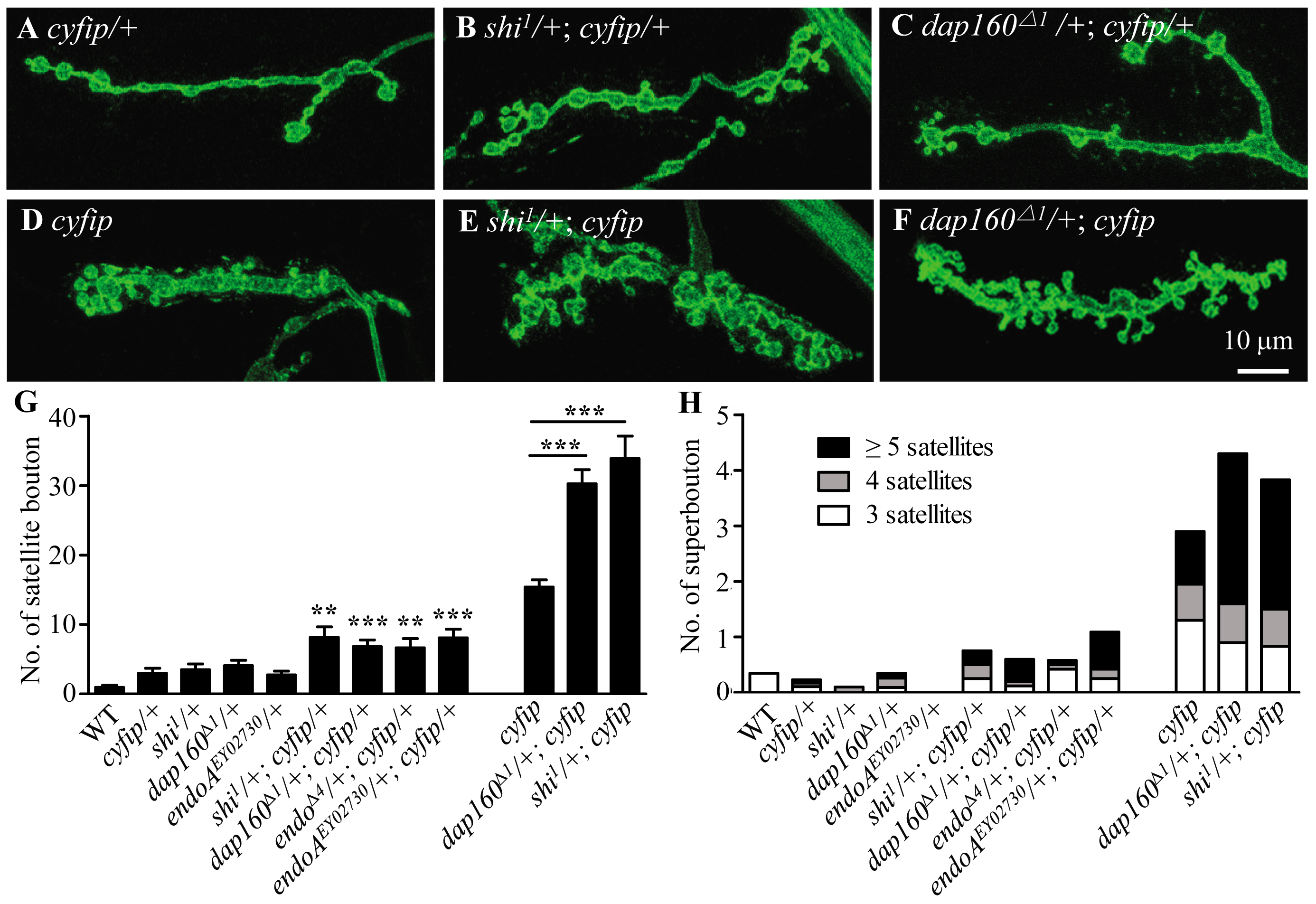

Supplement: Figure S4 — Satellite boutons in cyfip mutants are dominantly enhanced by endocytic mutants. (A–F) Endocytic mutants shi1 (a temperature sensitive allele), dap160Δ1 (a null or severe hypomorph), endoAΔ4 (a null allele), and endoAEY02730 (a hypomorph) (all from the Bloomington Stock Center) were used for genetic interaction analysis. Representative images of NMJ4 synapses labeled with anti-HRP from cyfip85.1/+ (A), shi1/+; cyfip85.1/+ (B), dap160Δ1/+; cyfip85.1/+ (C), cyfip85.1 null mutants (D), shi1/+; cyfip85.1 (E), and dap160Δ1/+; cyfip85.1 (F). Scale bar, 10 µm. (G, H) Statistical results of the number of satellite boutons (G) and superboutons (H) from different genotypes at 25°C. n≥16 for each genotype. * p<0.05, ** p<0.01, and *** p<0.001; error bars indicate SEM. (TIF) [file pgen.1003450.s004.tif]
